# Revisiting gap locations in amino acid sequence alignments and a proposal for a method to improve them by introducing solvent accessibility

**DOI:** 10.1002/prot.23011

**Published:** 2011-02-10

**Authors:** Atsushi Hijikata, Kei Yura, Tosiyuki Noguti, Mitiko Go

**Affiliations:** 1 Division of Biological Science, Graduate School of Science, Nagoya UniversityFuro, Chikusa, Nagoya 464-8602, Japan; 2 Computational Biology, Graduate School of Humanities and Sciences, Ochanomizu University2-1-1 Otsuka, Bunkyo, Tokyo 112-8610, Japan; 3 Center for Informational Biology, Ochanomizu University2-1-1 Otsuka, Bunkyo, Tokyo 112-8610, Japan; 4 Oasis Daini Hospital2-3-30 Higasi-Tsurusaki, Ooita 870-0103, Japan; 5 Department of Bioscience, Faculty of Bioscience, Nagahama Institute of Bio-Science and Technology1266 Tamura, Nagahama, Shiga 526-0829, Japan; 6 Tokyo Medical and Dental University1-5-45 Yushima, Bunkyo, Tokyo 113-8510, Japan; 7 Research Organization of Information and Systems4-3-13, Toranomon, Minato, Tokyo 105-0001, Japan

**Keywords:** ALAdeGAP, amino acid sequence alignment, comparative modeling, position dependent gap penalty, solvent accessibility

## Abstract

In comparative modeling, the quality of amino acid sequence alignment still constitutes a major bottleneck in the generation of high quality models of protein three-dimensional (3D) structures. Substantial efforts have been made to improve alignment quality by revising the substitution matrix, introducing multiple sequences, replacing dynamic programming with hidden Markov models, and incorporating 3D structure information. Improvements in the gap penalty have not been a major focus, however, following the development of the affine gap penalty and of the secondary structure dependent gap penalty. We revisited the correlation between protein 3D structure and gap location in a large protein 3D structure data set, and found that the frequency of gap locations approximated to an exponential function of the solvent accessibility of the inserted residues. The nonlinearity of the gap frequency as a function of accessibility corresponded well to the relationship between residue mutation pattern and residue accessibility. By introducing this relationship into the gap penalty calculation for pairwise alignment between template and target amino acid sequences, we were able to obtain a sequence alignment much closer to the structural alignment. The quality of the alignments was substantially improved on a pair of sequences with identity in the “twilight zone” between 20 and 40%. The relocation of gaps by our new method made a significant improvement in comparative modeling, exemplified here by the *Bacillus subtilis* yitF protein. The method was implemented in a computer program, ALAdeGAP (ALignment with Accessibility dependent GAp Penalty), which is available at http://cib.cf.ocha.ac.jp/target_protein/. Proteins 2011; © 2011 Wiley-Liss, Inc.

## INTRODUCTION

Most of the proteins perform their function after forming their three-dimensional (3D) structures. Knowledge of protein 3D structure is, therefore, essential for understanding the mechanisms of protein function in atomic detail.^1^ Consequently, a large number of protein structures have been determined systematically by structural genomics projects,^2^,^3^ with the goal of elucidating the function of proteins known from genome sequences. The number of experimentally determined protein 3D structures is now over 60,000.^4^ The number of amino acid sequences derived from genome sequences, however, is over 6,000,000, much larger than that of protein 3D structures.^5^ Experimentally determining all of these protein 3D structures would take a prohibitively long time, thus computational study of protein 3D structures is expected to help to meet this need. Template-based comparative modeling, based on protein family classification, is currently the most promising method for narrowing the gap between the number of structure known and unknown proteins.^6^,^7^

Comparative modeling technique consists of several component methods: a method for finding the best template structure; a method for high-accuracy alignment; and a method for accurately deducing side-chain conformation.^8^ Current techniques for comparative modeling have been significantly improved but are still rarely able to generate a model that is comparable in quality with structures determined by X-ray crystallography. This is especially true for cases in which the template and target structure share low sequence identity. The accuracy of deducing side chain conformations has been increased by the introduction of rotamer libraries, especially those that contain dihedral angle-dependent chi-angle distributions with sophisticated statistics.^9^ It is becoming possible to precisely predict the configuration of side chains at the active site; this is especially important in order for model structures to be useful in ligand docking.^10^ Methods for identifying the best template improved significantly with the advent of the 3D−1D method,^11^ followed by the PSI-BLAST^12^ and profile−profile methods.^13^–^15^ Using these methods, we can reliably find an appropriate template, but there still is substantial room for improving the alignment, that is, the residue−residue correspondence between the template and the target sequences.^16^ Especially when two sequences are only distantly related, then the sequences have undergone a relatively large number of insertions and deletions, and hence finding corresponding residue pairs becomes difficult.

Efforts dedicated to improving alignment quality have focused primarily on improving the substitution matrix. Some approaches have attempted to build a general substitution matrix that depends on the protein environment,^17^ whereas others have introduced a position-specific substitution matrix or profile,^18^–^24^ and others have combined sequence alignment with 3D structural alignment.^25^ Adjustment of the locations of insertions and deletions (hereafter called gaps) was also attempted in an effort to improve the quality of alignment. Typical alignment methods incorporate the affine gap penalty function.^26^ Parameters in the equation for the affine gap penalty were optimized to best recall the pairwise alignment obtained from 3D structure comparison.^27^ It is assumed that the correspondence of residues in the best sequence alignment should be the same as the correspondence obtained by comparison of the 3D structures. This assumption is especially reasonable when the sequences are the template and the target for comparative modeling.

When aligning amino acid sequences for the purpose of comparative modeling, the 3D structure of the template protein is known by definition; hence, the structural information can be reflected in the gap penalty. Lesk *et al.*^28^ first focused on this issue by observing that, based on the structural comparison of human and lupin globin proteins, gaps rarely occur in the interior of helical regions of proteins. Those authors introduced a variable gap penalty that was higher in the interior of helices and strands than in regions that lacked such secondary structures; this approach improved the resulting alignment. The rigorous test of the relationship between gap location and protein 3D structure was first performed by Zhu *et al.*^29^ on 15 protein families; a linear relation was observed between side-chain accessibility and the frequency of gaps. They used this relation and the relation that gaps are underrepresented in regions of defined secondary structure^28^ to improve COMPARER, a 3D structure comparison program.^30^ Madhusudhan *et al.*^31^ applied these relations to variable gap penalty and increased the accuracy of alignment from 81.0 to 84.5% in a dataset of 238 sequence pairs with known 3D structures. Qiu and Elber^32^ developed a new gap penalty calculation method in SSALN; this gap penalty depended on 12 different structure types, according to the predicted secondary structure, predicted relative solvent accessibility for each residue of the target sequence, and the real values of the secondary structures and relative accessibility for each residue in the template sequence.

Even after all these past efforts, the quality of the protein model still falls below the satisfactory level. Kopp *et al.*^33^ pointed out in a summary of CASP7 (the seventh Critical Assessment of Techniques for Protein Structure Prediction) that alignment was by no means a solved problem and constituted a major bottleneck in comparative modeling. The CASP8 assessment of template-based modeling identified a major challenge: locating an accurate place and conformation for loops inserted into the 3D structure of template proteins.^34^

Here, we re-evaluated the premise of gap location in protein 3D structures, based on a large protein dataset, and found that the distribution of gaps in protein 3D structures differed from those reported previously. We examined the pure contribution of our new finding to the improvement in sequence alignment by implementing a new gap penalty equation into a simple pairwise alignment method. We found that the new method outperforms most of the conventional alignment methods. Our new program will help to improve the quality of comparative modeling by providing a better alignment between template and target amino acid sequences. The software is available at http://cib.cf.ocha.ac.jp/target_protein/.

## METHODS

### A dataset of superposed protein 3D structures

Gap locations were assigned by superposing a pair of homologous protein structures. The homologous protein pairs were taken from each family in SCOP 1.69 dataset.^35^ We used a single domain protein in the SCOP families included in either of the all alpha, all beta, or alpha and beta (a/b, a+b) protein classes to minimize the technical difficulty of superposing two structures. Proteins with coordinates for fewer than 60 residues were not included in our dataset. If multiple 3D structures of proteins with identical sequence existed, then the structure determined with the best resolution was taken as the representative. In each SCOP family, protein pairs were chosen so as to maximize the total number of pairs and minimize the sequence identity within each pair. Each pair of proteins was then superimposed using Combinatorial Extension,^36^ and the location of gaps was determined based on the structural alignment. Pairs without gaps were discarded. Ultimately, we obtained 18,019 superimposed protein pairs.

### “Gap accessibility” and gap frequency against the accessibility

**Figure 1:**
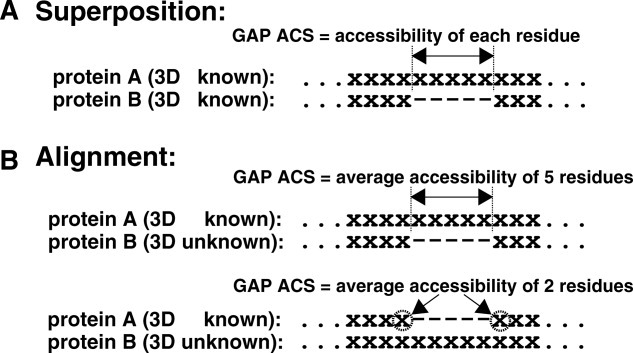
A method to obtain the accessibility (ACS) of gaps. (**A**) Residue-wise gap accessibility is given by the accessibility of the residues in the gap. (**B**) In the alignment, if the 3D structure of an insertion segment is known, then the gap accessibility can be directly calculated. If the 3D structure is unknown, then it is calculated as the average of accessibilities of residues flanking the deletion.

“Residue-wise gap accessibility” was defined by the accessibility of each residue aligned in the gap region [Fig. 1(A)]. We herein named the residue-wise gap accessibility the “gap accessibility.” The accessible surface area of an atom was calculated using the method of Shrake and Rupley,^37^ implemented in an in-house program. The accessibility of each residue was calculated based on the method described by Go and Miyazawa,^38^ Gap accessibility was categorized into bins of width *w* with 0.05, and *N*_*i*_, the number of gaps with a gap accessibility in each bin, was counted. Then *g*_*i*_, the frequency of gaps in each accessibility bin *i*, was calculated by,


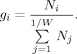
(1)

To compare *g*_*i*_ in different bins, the value should be normalized by the frequency of residues in each bin, *f*_*i*_, which is,


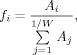
(2)

where *A*_*i*_ is the count of residues in accessibility bin *i*. 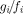 is the odds ratio on finding a gap in bin *i*. A rule applicable for building a sequence alignment was then deduced as an equation by observing the relationship between the accessibility and the gap odds ratio.

### Implementation of the gap penalty into standard sequence alignment method

We developed a program for pairwise amino acid sequence alignment based on the assumption that one of the sequences has a known 3D structure (template) and the other does not (target). A pairwise alignment by dynamic programming was implemented as described by Isaev,^39^ using the BLOSUM62 amino acid substitution matrix^40^ adjusted to have non-negative elements. The affine gap penalty^26^ was used, and two parameters (gap opening and extension penalties) were adjusted by maximizing the number of correctly aligned residue pairs. Structural alignments were considered as correct alignments. The gap opening penalty was set to 13, and the gap extension penalty to 1. The program was then modified to take into account the residue accessibility in gap opening penalty based on the gap calculation equation given in the previous section. All possible gap opening penalties were precalculated and stored in a gap matrix before commencing a dynamic programming calculation. Gap accessibility was calculated as shown in Figure 1(B). When the gap region was a deletion of the template protein, then the gap accessibility was the average of the accessibilities of the deleted residues. When the gap region was an insertion to the template protein, then the gap accessibility was the average of the accessibilities of two flanking residues. The coefficients in the gap equation were determined numerically by maximizing the number of residue pairs in the sequence alignments that matched residue pairs in the structural alignments. The number of structural alignments used for parameter fitting was reduced from the original dataset built for the investigation of the gap location, by eliminating pairs with more than 90% and less than 20% sequence identities.

### Comparison of the method with the conventional ones

We used three types of scores to compare the performance of our alignment method with conventional ones. The first is the Q-score, defined by Pei and Grishin,^24^ which evaluates the overall alignment quality. The Q-score is the number of correctly aligned residue pairs in the sequence alignment divided by the total number of aligned residue pairs in the structural alignment; thus, its value is between 0 and 1. The second score is an evaluation of the accuracy in locating an insertion segment (*I_s_*). 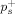 is the number of correctly assigned insertion segments in the sequence alignment (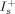) divided by the total number of the assigned segments (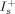 + 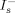). The correctly assigned segment is defined by an overlap of the segments; when the assigned segment and the segment in the structural alignment overlap by at least one residue, then the segment is defined as correct. The third score evaluates the accuracy in locating an insertion point (*I_p_*). In this score, a three-residue window is set around the insertion point identified by a structural alignment; if the insertion point identified by the sequence alignment is located in this window, then it is assigned as correct. 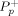 is the number of correctly assigned insertion points in the sequence alignment (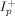) divided by the total number of assigned insertion points (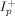 + 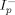).

From the viewpoint of comparative modeling, alignment can be recognized as a method for gap prediction. Accurate prediction of *I_s_* and *I_p_* is then a prerequisite for modeling. Taking the modeling procedure into account, the accuracy of the model is best measured with correctness and no over-assignment of *I_s_* and *I_p_*. We quantified this idea in the following equations;


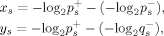
(3)

where 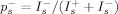, 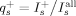, and 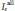 is the number of real insertion segments in the structural alignment. The ideal alignment for comparative modeling should have *x*_*s*_ << 0, because correctly assigned insertion segments should outnumber incorrectly assigned segments, and *y*_*s*_ ≈ 0 or at least *y*_*s*_ ≤ 0, because assignment of too many insertion segments significantly hampers the comparative modeling process. Similar equations can be applied to *I_p_*;


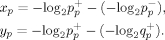
(4)

We compared the accuracy of alignment methods for comparative modeling based on Q-score and Eqs. (3) and (4).

### Implementation of multiple sequence alignment method

In our program, a progressive multiple sequence alignment method^27^ was implemented. A guide tree was first built based on Kimura's distance,^41^ calculated from pairwise sequence identity, and the alignment was built progressively from the leaves to the root of the tree. If one of the sequences being aligned had 3D structure information, then the accessibility-dependent gap penalty was used; if not, the fixed gap penalty was used.

## RESULTS AND DISCUSSION

### Gap frequency against “gap accessibility”

**Figure 2:**
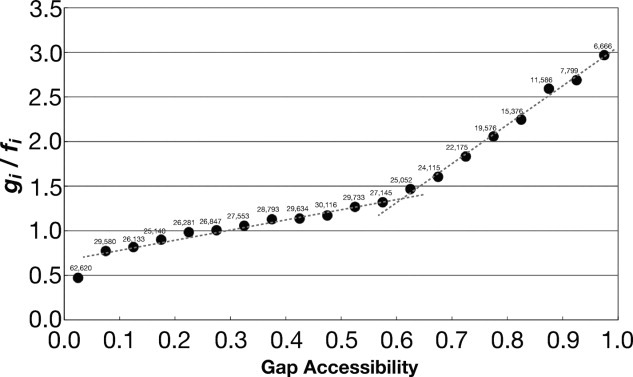
Odds-ratio of a gap as a function of gap accessibility. The number above the dot is the number of gaps (the number of residues aligned against a gap) in each accessibility bin. Standard deviation of each plot was determined by a bootstrap procedure with 1000 resamplings and it turned out to be smaller than the radius of each dot.

The relationship between gap accessibility and gap frequency was revisited using a large dataset based on SCOP 1.69,^35^ from which we extracted 18,019 superposed protein pairs. Figure 2 shows the odds of a gap as a function of the accessibility of residues aligned to the gap (gap accessibility). We used bootstrap method with 1000 resamplings to estimate the standard deviations of each plot, and found that the standard deviations were smaller than the radius of each dot on the graph. The distribution can be approximated by a combination of two straight lines, as shown by the dotted lines in Figure 2. The line from gap accessibility of 0.0 to 0.6 has a less steep gradient than the second line from 0.6 to 1.0. Gap accessibility of 0.6 seems to be a critical point where the relationship between gap frequency and gap accessibility changes. The similar trend in gap accessibility was found even when we divided the data in different sequence identity ranges (data provided in the Supporting Information). The physicochemical meaning of this critical point is unknown. So far, we could not find any obvious relationship between gap accessibility and, for instance, secondary structure that may account for the observed change in slope in figure. We speculate that this change in gradient may correlate with a change in the packing density of the residues in proteins.

The first line crosses *g*_i_/*f*_*i*_=1.0 at a gap accessibility of ∼0.3, which means that an accessibility of 0.3 is the point where gap preference switches: gaps are underrepresented between accessibility of 0.0 and 0.3, and overrepresented at accessibilities greater than 0.3. Go and Miyazawa^38^ demonstrated that the variability of amino acid residues in the process of protein evolution differs around an accessibility of 0.27. Specifically, they showed on eight representative proteins that residues which remained invariant over the course of evolution were overrepresented in sites where accessibility was no more than 0.27. Both their result and ours indicate that structural changes in the interior of protein (accessibility less than ∼0.3) are significantly suppressed during evolution, presumably due to constraints required to maintain protein 3D structures.

For the gap penalty calculation in sequence alignment, it is preferable to obtain the gap penalty using a single continuous equation for accessibility. We fit the plot with a linear and logarithmic regression lines. Linear regression of the plots resulted in *y* = 2.25*x* + 0.35 (residual error = 0.96), whereas natural log regression of the plots resulted in log *y* = 1.55*x* − 0.50 (residual error = 0.21). The distribution of the frequency of the gap can be reasonably expressed via a logarithmic equation. The gap penalty should be in inverse relation to the gap frequency; hence, we used an exponential equation to deduce the gap penalty for sequence alignment.

The logarithmic relationship between gap accessibility and gap odds ratio has not been previously reported. Zhu *et al.*^29^ was the first to analyze the relationship between accessibility of residues and the frequency of gaps and showed a linear relation between them. The discrepancy may stem from differences in the size and types of dataset used. Our dataset contains many types of proteins from a large number of protein families.

### Gap penalty equation

We found the logarithmic relationship between gap location and gap accessibility in the previous section. To reflect this relationship to the gap penalty, which should be the inverse of the gap frequency, we used the following equation for the accessibility-dependent gap opening penalty *G*;


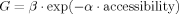
(5)

where accessibility was calculated as shown in Figure 1(B), and both α and β are parameters fit to maximize Q-score, the recall rate of structural alignments. The extension gap penalty was kept at 1.

**Figure 3:**
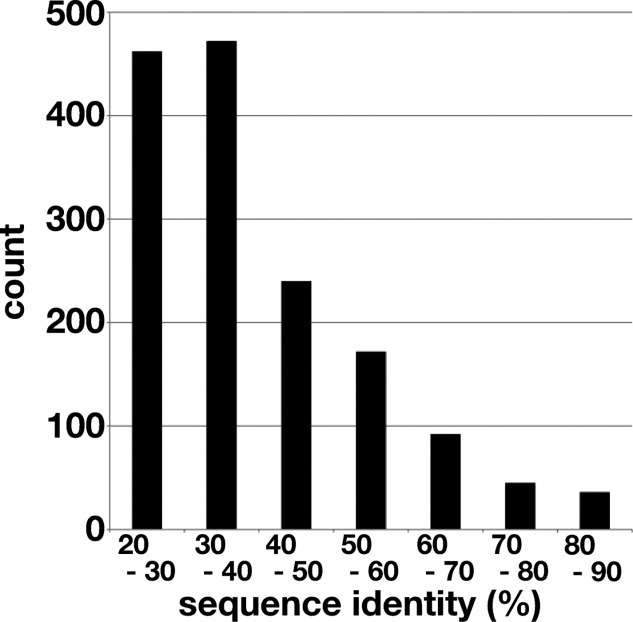
Distribution of sequence identity in the protein pair dataset used for gap penalty parameter fitting. The horizontal axis indicates bins for identity ranges. Sequence identity was calculated based on the correspondence of residues assigned by structural alignment. All protein pairs are shown in the Supporting Information.

The dataset we used for parameter fitting was a subset of the dataset we observed the relationship between gap frequency and accessibility. From the original dataset with 18,019 protein pairs, we selected 1519 pairs. The pairs were selected to avoid multiple appearances of the same protein, and to have lower sequence identity. The distribution of amino acid sequence identity in the selected dataset is shown in Figure 3; the whole list of pairs of proteins with sequence identity is provided in the Supporting Information. We checked the frequency of gaps against the accessibility in this small dataset and found similar characteristics to those we discussed in the previous section (data not shown). The new dataset does not contain a pair with identity less than 20% (Fig. 3).

### Gap penalty parameter fitting

A brute force parameter search was performed in the range 1.0 ≤ α, β ≤ 39.0 with an interval of 1.0. This search revealed that α = 2.0 and β = 33.0 achieved a Q-score of 0.910. Q-score was sensitive to α, because all the parameter sets with a Q-score of 0.91 had α = 2.0. We then further searched for the parameter set in the range 2.0 ≤ α ≤ 3.0 and 31.0 ≤ β ≤ 33.0 with an interval of 0.1 and found that α = 2.1 and β = 32.8 were the best set, with Q-score = 0.911. This score means that 374,784 matches in the alignment are correct, out of the 411,337 matches in 3D structure comparison. The original implementation of the alignment with affine gap penalty resulted in a Q-score of 0.870. We also implemented a gap penalty with a linear relationship with accessibility and obtained a maximum Q-score of 0.902. Hence, the introduction of gap penalty with exponential relation against the accessibility improved the alignment by about 4%. The percentage seems small, but 4% corresponds to ∼15,000 residue matches in 1519 protein pairs. Improvement of the match in ∼10 residues (=15,000/1519) in one protein pair can coincide with an improvement of loop location by relocating a gap in the alignment. The impact of this is exemplified in the last section. The program with the best parameters was named ALAdeGAP (ALignment with Accessibility dependent GAp Penalty), and it is freely available at http://cib.cf.ocha.ac.jp/target_protein/.

### Performance comparison: comparison with ClustalW and MAFFT

**Figure 4:**
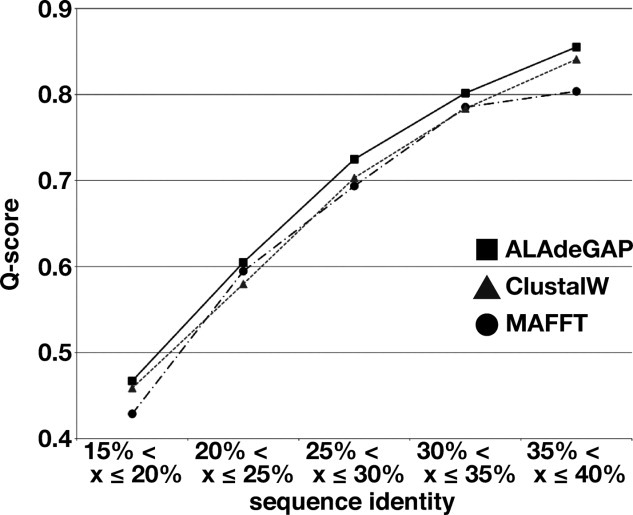
Comparison of the alignment performance among ALAdeGAP (our newly developed method), ClustalW^42^ and MAFFT.^21^

ClustalW^42^ and MAFFT^21^ are two of the most widely used sequence alignment methods among molecular biologists; hence, we first compared the performance of ALAdeGAP to those two methods. The other reason we chose those two alignment methods is that those programs can be used for pairwise alignment and can be run without sequence profiles. The comparison, therefore, can be made purely on the basis of adjusting the gap penalty. For the performance comparison, we used protein pairs which were not included in SCOP 1.69, because the parameters in ALAdeGAP were adjusted using SCOP 1.69 and using a protein pair in SCOP 1.69 for the performance comparison blurs objectivity of the test. In addition, ALAdeGAP concentrates on improving the location of gaps; alignments with many gaps tend to have low sequence identity. We, therefore, compared performance on sequence pairs of low sequence identity. The number of protein pairs in the dataset were as follows: 66 pairs with identity of 15% < *x* ≤ 20%, 75 pairs with identity of 20% < *x* ≤ 25%, 66 pairs with identity of 25% < *x* ≤ 30%, 72 pairs with identity of 30% < *x* ≤ 35%, and 59 pairs with identity of 35% < *x* ≤ 40% range. The result of Q-score comparison is shown in Figure 4. In all these ranges, ALAdeGAP outperformed the other two methods. The difference may seem marginal, but note that ALAdeGAP adjusts the location of gaps, but improvement in the gap location does not dramatically improve Q-score, because this metric reflects the number of residue−residue pairs rather than the difference in gap location. The significance of the improvement of the alignment by adjusting gap location can be observed in individual cases of alignment; we will describe a specific example in the last section. Note that MAFFT has the worst performance in the 35−40% range; this may be a consequence of using this method for pairwise alignment, an application that was not anticipated by its developers.

### Performance comparison: comparison with PROMALS on SABmark

PROMALS is one of the best multiple sequence alignment programs for distantly related sequences.^24^ The performance of ALAdeGAP and PROMALS was compared on SABmark benchmark, a set of paired protein sequences that covers the entire known fold space.^43^ SABmark provides a “super-family” set and a “twilight-zone” set. The original idea of the SABmark benchmark set is to compare the accuracy in assigning residue-to-residue correspondence in the “super-family” set and is to compare the accuracy in detecting remote homologues in the “twilight-zone” set. ALAdeGAP is not aiming for remote homologue detection, and hence we only used the “super-family” set for this benchmark.

**Figure 5:**
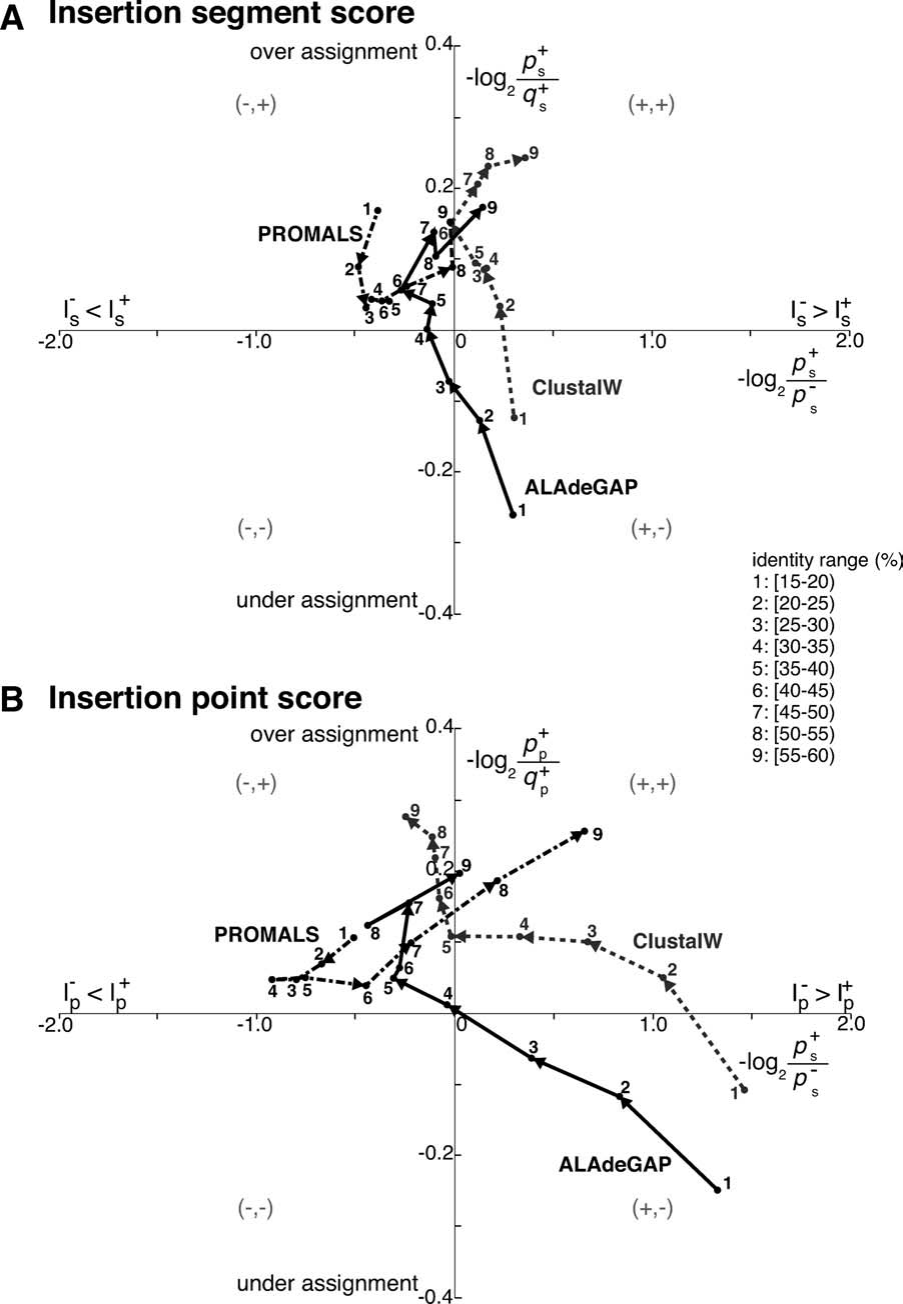
A performance comparison among ALAdeGAP, PROMALS,^24^ and ClustalW^42^ on the SABmark benchmark superfamily set.^43^ The performance was compared based on *Is* (**A**) and *Ip* (**B**). See Eqs. (3) and (4) in the Methods section for the definition of each score. Pairs of protein superfamily sequences in the SABmark superfamily set were classified into five-percent sequence identity bins, from 15−20% to 55−60%. The performances of the three different alignment methods were tested on sequence pairs in each bin, and performance scores were plotted. The dots are connected in ascending order of sequence identity. Note that a method with a line running into or close to the (−,−) region is considered to be the best.

The result of the comparison based on Eqs. (3) and (4) is shown in Figure 5. The performance of ClustalW^42^ is also shown. The horizontal axis is equal to the logarithm of the number of correct gap assignments divided by the number of incorrect gap assignments. A negative value indicates that the number of correct gap assignments exceeds the number of incorrect assignments. The vertical axis is equal to the logarithm of the ratio of correctly assigned gaps in all of the gaps assigned by sequence alignment, divided by the ratio of correctly assigned gaps to the total number of real gaps assigned by structural alignments. A positive value indicates over-assignment, and a negative value indicates under-assignment. Zero is the best value on the vertical axis and negative is better than positive for the purposes of comparative modeling. Too many (and mostly incorrect) assignments of gaps in the protein 3D structure may hamper the modeling procedure. The best alignment for comparative modeling needs to have as many as correct gap assignments; hence the alignment should reside in or close to the area where both values are negative. The protein pairs in the benchmark set were categorized into sequence identity bins, and performance was compared on the set of protein pairs in each bin. Each bin was numbered from 1 to 9 in ascending order of sequence identity; each dot in the graph corresponds to a result of comparison in each bin and is numbered accordingly.

The plots for ALAdeGAP (solid black line), and ClustalW (broken grey line) started at lower right in both insertion segment (*I_s_*) score (A) and insertion point (*I_p_*) score (B), and ran to the upper right in (A) and upper left in (B). The general trends of the both plots are similar. The trend of the plot for PROMALS (dotted black line) is different from the other two: the plot generally stays in the upper left region in (A) and (B). The interpretation of the lower right region (+,−) is that the number of false gap assignments exceeds that of true gap assignments, but the number of assigned gaps is less than the number of the real gaps. The interpretation of the upper right region (+,+) is that the number of false gap assignments exceeds that of true gap assignments, and the number of assigned gaps is greater than the number of real gaps. The interpretation of the upper left region (−,+) is that the number of true gap assignments exceeds the number of false gap assignments, but the number of assigned gaps is greater than the number of real gaps. Note that ALAdeGAP is the only method that goes through or runs close to the (−,−) region, where the number of true gap assignments exceeds the number of false gap assignments and the number of assigned gaps is less than the number of real gaps.

Figure 5 shows that neither the conventional methods nor ALAdeGAP can achieve the best alignment method for comparative modeling, but ALAdeGAP is closest to optimal. PROMALS does have good value on the horizontal axis but has a tendency to over-assign gaps in all sequence identity ranges. ALAdeGAP runs into or close to the (−,−) region for pairs with sequence identity between 20 and 40% in *I_s_*, and runs close to (−,−) region for pairs in a similar identity range in *I_p_*. ALAdeGAP is well suited for alignment for the purpose of comparative modeling of a sequence pair in this range. In the parameter fitting dataset, we put stress on increasing the number of data in this range, because improvement in comparative modeling in this range of sequence identity is mostly in need. The apparent good performance between 20 and 40% identity range may be related to this abundance of aligned sequences in this particular range (Fig. 3).

In a comparison between PROMALS and ALAdeGAP based on Q-score, PROMALS outperformed ALAdeGAP (data not shown), but this is because of the difference in the information used by each program. PROMALS incorporates sequence information obtained by PSI-BLAST.^12^ The performance of PROMALS is far better than ALAdeGAP when the sequence identity of the protein pair is less than ∼20%. ALAdeGAP is not based on profile, whereas PROMALS makes use of this information; this causes a difference in performance in the low sequence identity range. The developers of PROMALS further improved the alignment by incorporating structural alignment into a multiple sequence alignment (PROMALS3D).^25^ As PROMALS3D directly incorporates information about multiple protein 3D structures, we did not compare the ALAdeGAP alignment with the PROMALS3D alignment; in comparative modeling, the target protein 3D structure would by definition never be known beforehand.

### Application of ALAdeGAP to hypothetical protein YitF

**Figure 6:**
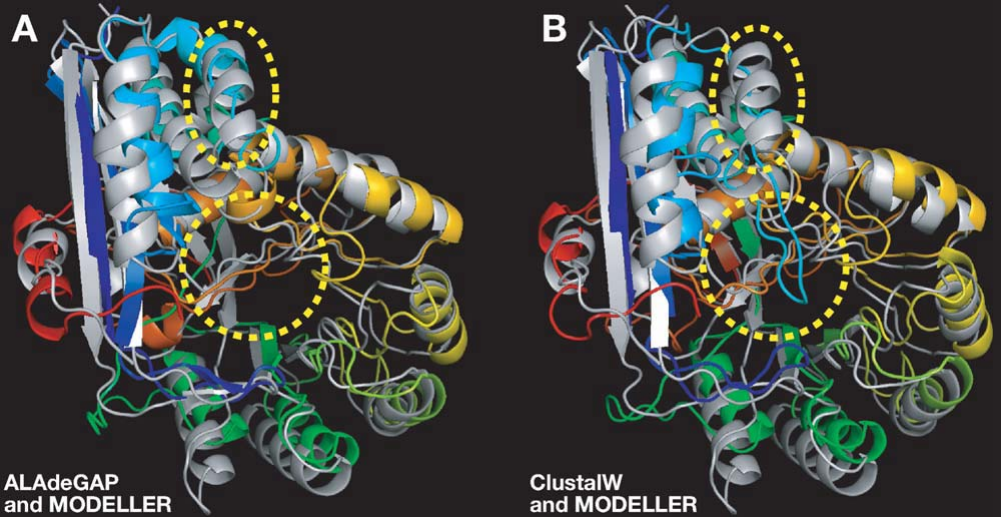
3D structure of *Bacillus subtilis* yitF based on ALAdeGAP alignment between yitF and *Escherichia coli* GlucD (A) and on ClustalW alignment between yitF and GlucD (B). Amino acid sequence identity is ∼19%. The model was built by MODELLER.^46^ In either figure, the colored chain is the modeled structure, and the white chain is the structure determined by X-ray crystallography. Yellow dotted circles emphasize the differences in both structures. The structure is viewed in the direction of the active site. In B, the active site is covered by an inappropriately modeled loop. The figure was drawn using PyMOL.^47^

Comparative modeling with improved gap location in the template−target alignment is expected to have higher chance of guiding protein function annotation in the right direction. In the *Bacillus subtilis* genome, there were about 2850 genes (70%) without known functions at the time of genome sequencing.^44^ The names of these genes are prefixed by “y” (“y” genes), and determination of their biological functions has been ongoing since their first annotation. YitF gene encodes a protein belonging to the enolase superfamily and annotated as mandelate racemase, but the function has not been verified. Possible orthologues of yitF only exist in the *Bacillus* genus; homologous proteins in other genera have low amino acid sequence identity, which implies that an accurate multiple sequence alignment is hard to obtain. We modeled the 3D structure of *Bacillus subtilis* yitF using *Escherichia coli* D-glucarate dehydratase (GlucD) 3D structure^45^ (PDB ID, 1jdf) as a template. A pairwise alignment was built using ALAdeGAP or ClustalW, and the structures were built with MODELLER^46^ (Fig. 6). The sequence identity was ∼19%. The most crucial difference between the two alignments was found at the sequence around the active sites. The 3D structure of *Bacillus subtilis* yitF was later determined in a structural genomics project (PDB ID, 2gdq) and we can assess the accuracy of the model. The difference in overall Cα root mean square deviations was slight, 4.8 Å for ALAdeGAP model and 5.5Å for ClustalW model. However, due to the inappropriate location of gaps in the alignment, the active site of the protein was covered by a loop in the ClustalW model, and one of the α helices was melted to a loop [Fig. 6(B)]. A prediction of the substrate for this enzyme based on the ClustalW model would therefore be misleading.

## CONCLUSION

We built ALAdeGAP, a new sequence alignment method for comparative modeling. The method is based on the characteristics of protein evolution, namely the gap (insertion and deletion of residues) occurs more frequently on the surface of protein 3D structures. We found that the relation between the frequency and accessibility of gap region is nonlinear. By incorporating this dependency, ALAdeGAP can improve the location of gaps in the alignment when the sequence identity is between ∼20 and ∼40%, a range in which standard methods tend to misplace gaps. We have already implemented our new method to enable multiple sequence alignment. The details of the application of the method will be explained elsewhere. Current threading methods also suffer from precisely locating gaps, namely determination of the precise boundaries of the different elements of secondary structure for the target sequence. Our finding here may indicate a possible benefit, when this gap affinity score could be properly incorporated onto the existing threading algorithms.
