# Supplementary material for: Revisiting gap locations in amino acid sequence alignments and a proposal for a method to improve them by introducing solvent accessibility
Source: Proteins. 2011 Feb 10;79(6):1868–77. doi: 10.1002/prot.23011 (PMC3110861; doi:10.1002/prot.23011)
Supplement: Supplementary file 1 [file prot0079-1868-SD1.pdf]

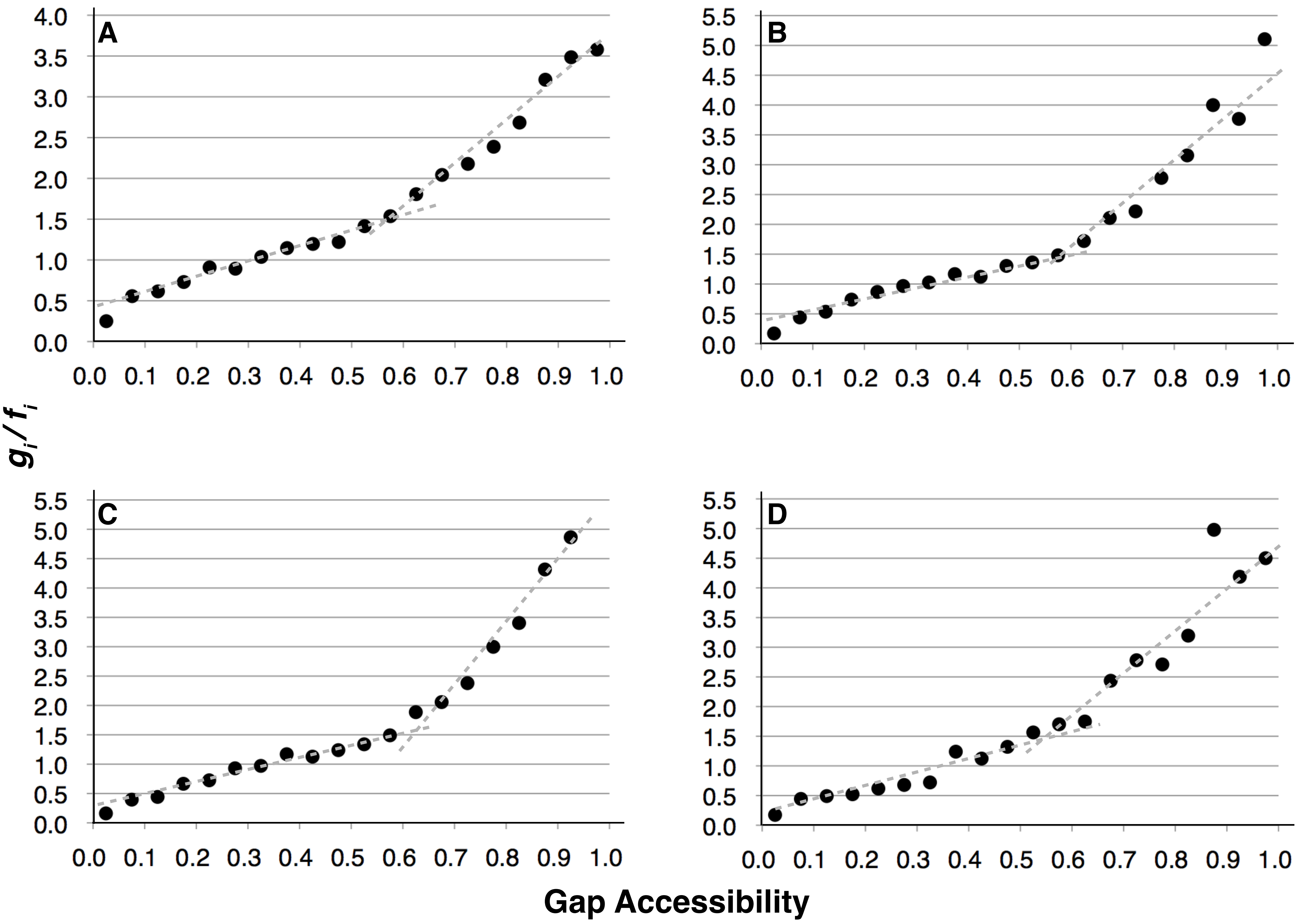

Supplementary Figure 1: Odds-ratio of a gap as a function of gap accessibility. These figures are the result of decomposing Figure 2 according to sequence identity. **A**: sequence identity between 20% and 30%, **B**: sequence identity between 30% and 40%, **C**: sequence identity between 40% and 50%, **D**: sequence identity more than 50%. Note that the trend discussed in Figure 2 is evident in each graph. Statistical stability of each plot is low, because the number of cases is quite small compared with the numbers in Figure 2.
