# Supplementary material for: Revisiting gap locations in amino acid sequence alignments and a proposal for a method to improve them by introducing solvent accessibility
Source: Proteins. 2011 Feb 10;79(6):1868–77. doi: 10.1002/prot.23011 (PMC3110861; doi:10.1002/prot.23011)
Supplement: Supplementary file 2 [file prot0079-1868-SD2.pdf]

PDBID1	PDBID2	identity
4np1b	1sy1a	89.7
1d3ga	1uumb	89.5
1u0fb	1n8tb	89.4
1gc6a	1e5wa	89.4
1exra	1ooja	89.1
1rr6a	1b8oa	89.0
1r2ra	1tph2	89.0
1hjoa	1qqoa	88.4
1l5da	1jk4a	88.3
1necd	1ds7a	88.0
1kxoa	1bbpa	87.8
1iuaa	1b0ya	87.8
1yaga	1atna	87.5
1umka	1i7pa	87.0
1rzkg	1rzjg	86.9
1dy1a	1bnla	86.6
1w6pb	1slcd	86.4
1dhka	1c8qa	86.2
1smyl	1hqmb	86.0
1qrdb	1d4aa	85.7
1fr5a	1aq3a	85.6
1ethc	1n8sa	85.3
1nq3c	1onic	85.1
1ngma	1volb	85.0
1tahd	1oilb	83.0
1rbla	1aa1l	82.9
1ucsa	1b7ia	82.8
1rihh	1ngqh	82.3
1y92a	1xptb	81.6
1g2ff	1a1fa	81.2
1khud	1u7va	81.0
1khob	1qmdb	81.0
1fmms	1barb	80.6
2tnfa	1tnfa	80.4
1gyca	1kyad	80.4
1ldtt	1v2wt	80.3
1a5db	1i5ia	79.8
1jota	1jaca	79.7
1xtcf	1pzih	79.6
1ht1a	1g3ka	79.2
1tfpb	1bm7a	78.3
1uvcb	1fk7a	78.0
1fnia	1v2kt	78.0
1rgya	1pi5b	77.7
1kcul	1aifl	77.1
1forh	1afvk	77.1
1qj4a	1dwoa	77.0
1figl	2pcpc	76.9
1eu1a	1e60a	76.9
6paxa	1mdma	76.0
1figh	1mreh	76.0
1jb0e	1qp2a	75.8
1offa	1qt9a	75.0
1ds6b	1rhob	74.5
9cgta	1pj9a	74.1
1uzrb	1kgna	74.1
1ydfa	1wvid	74.0

2atcb	1skub	73.9
1a0dd	1a0ca	73.9
1f1ma	1ggqd	73.3
1rkrd	1ilud	73.2
1trnb	1v2nt	73.1
2dldb	1j49b	72.7
1h8xa	1b6vb	72.5
1dt6a	1og5b	72.5
1do2a	1g41a	72.3
1hqsb	1pb3a	72.0
1us7a	1uyma	71.8
1lk3i	1il1a	71.7
1gaqb	1rfkb	71.7
2igfh	1emth	71.6
1d9va	1o7tg	71.5
1q3ac	1umta	71.2
1kb5h	1jnlh	71.2
1brmc	1pqud	71.2
1aosb	1auwd	70.9
1d1ta	1ye3a	70.8
1bh6a	1to2e	70.8
1tpya	1kp9a	70.2
1jl2c	1f21a	70.2
1hsla	1lahe	70.2
1uqxa	1gzta	69.9
1s9vb	1klgb	69.9
1mgwa	1boxa	69.9
1ieha	1sjva	69.7
1t0aa	1vhaa	69.5
1liub	1f3xh	69.5
1l4ia	1kiug	69.5
1u0ub	1d6ib	69.3
1ywtb	1a37a	69.2
1srva	1dkdd	69.0
1rfzd	1y9id	69.0
1neza	1k8da	68.8
1hl7b	1a7ua	68.8
1vcqa	1wykd	68.5
1gt0d	1hrya	68.5
1uizd	1ca7a	68.4
1m5sd	1m5hh	68.4
1dxib	3xima	68.2
1frvd	1h2rl	67.7
1diea	2ximd	67.5
1fxid	1qoab	67.4
1ev1l	1d4m1	67.2
1yifd	1y7ba	67.1
1lj0a	1hkoa	67.1
1bi9a	1ag8d	67.1
1f56a	1ws7a	67.0
2bpa1	1gff1	66.9
1lk3m	1fnsl	66.5
1cf8l	1nbvl	66.5
1lv7a	1ixza	66.4
1b0wa	1g7la	66.4
1cp9a	1ai4a	66.3
2crka	1qk1h	66.2
1p6ka	3nosb	66.2

1mlwa	1dmwa	66.2
1iaih	1aifh	66.0
1c72d	3gtud	65.7
1l0ba	1jnxx	65.5
1qqla	1q04b	65.4
1e3dc	2frvs	65.4
1r45c	1g24a	65.3
1i85d	1dqtd	65.2
2bvwb	1qk2b	64.9
1nwpb	1cc3b	64.8
1mzya	1aq8a	64.8
7hsca	1dkyb	64.6
1i1ac	1oqoa	64.6
1jk8b	1fngb	64.5
1mc3b	1fxoa	64.4
1cp9b	1jx9b	64.4
1sega	1t7ea	64.1
1e91a	1s5rb	64.1
1ci3m	1cfma	64.1
1udna	1oypa	63.8
1ie7c	1krbc	63.8
1i4kc	1mgqg	63.8
1f66g	1aoic	63.8
1cicb	1rivh	63.7
1mo5a	1u98a	63.4
1gjha	1pq1a	63.4
1dqdh	1pskh	63.3
1ob0a	1ud3a	63.2
1mh5h	1mhhd	63.1
1e6yd	1e6va	62.9
1omya	1kv0b	62.3
1m6wb	1n8kb	62.2
1i7ga	2prga	62.2
1d4da	1qjda	62.1
1r03a	1mfrx	62.0
1ia5a	1nhcf	62.0
1aotf	1o4ra	62.0
1te6b	2oneb	61.8
1sdsb	1pxwb	61.8
1mrdh	1xf3b	61.7
1pq3b	1p8nc	61.6
1bt0a	1yd8v	61.6
1r1a2	1vrh2	61.4
1e0ha	1unkd	61.2
2bo9c	1m4la	61.1
1pxwa	1rlgb	61.0
1c9na	1v5ia	61.0
1isna	1ni2b	60.8
1ay1h	1fj1d	60.6
1fvaa	1ff3a	60.5
1s3kl	1fj1c	60.4
1g5wa	1pmpa	60.3
1dz0a	1ilsd	60.2
1xa0a	1y9ef	60.1
1vbpa	1ws5e	60.0
1huea	1b8za	60.0
1b4wa	1tp2b	60.0
1b4jl	1seql	60.0

1v7mi	1bgxh	59.9
1ka5a	1sphb	59.8
1gmzb	1a2aa	59.8
1b8ka	1sg1b	59.8
1f2db	1tzmc	59.7
1rxva	1mc8b	59.6
1sqea	1xbwd	59.4
1hixb	1h1ab	59.4
1pvab	1s3pa	59.3
1fl5b	1nldh	59.3
1a6da	1q3rd	59.3
1lx5a	1es7c	59.2
1qfra	1kklj	59.1
1ne7b	1cd5a	59.0
1ikfh	1mnuh	59.0
1yv9b	1ys9a	58.9
1p0cb	1u3tb	58.9
1fhfa	1atja	58.9
1exsa	1gxaa	58.9
1eo6a	1kota	58.9
1efua	1tuic	58.9
1to5d	1azva	58.6
2stta	1bc7c	58.5
1npbb	1nnrb	58.5
1ahwb	1i7zd	58.5
1u11b	1d7aa	58.4
1miml	1gpom	58.4
1qoia	2biux	58.3
1iq4a	1mjib	58.3
1a4ka	1k4cb	58.3
1a7ge	1f9fd	58.2
1frfl	1e3dd	58.1
1f1cb	1w5cv	58.1
1bxng	1gk8e	58.1
1a6ya	1cita	58.0
2phla	1uikb	57.9
1qwva	1gm0a	57.9
1i4aa	1haka	57.9
1h8ob	1d6vl	57.8
1e6oh	1tzhh	57.8
1ib6a	1mldd	57.7
1gjib	2ramb	57.6
3ayka	1hova	57.5
1qgjb	1qo4a	57.4
1itka	1sj2b	57.4
1hbof	1e6yf	57.4
1cj6a	1fbiy	57.4
1ru7f	1hgdb	57.3
1wppb	1wpna	57.2
1e6yb	1e6vb	57.2
1xmpa	1qcza	57.1
1rn7a	1r4ch	57.1
1ncja	1ff5a	57.1
1bz7a	1bafl	57.1
1a4kl	1kb5l	56.9
1foba	1hjud	56.8
1fhla	1hjqa	56.6
1rrvb	1pn3a	56.5

1mmfg	1diog	56.5
1g73d	1tw6b	56.5
1xf6c	1cpcl	56.4
1iu8b	1a2za	56.4
1gq2p	1gz3d	56.4
1cp3b	1i4ob	56.4
1a5ca	1fbad	56.4
1kzza	1ca4a	56.3
1xr6a	1xr5a	56.2
1wx0f	1vpxa	56.2
1w5fb	1w59a	56.2
1iica	1nmtb	56.1
1kq8a	2hdca	56.0
1bbjl	1nldl	55.9
1rhhd	1uz8b	55.8
1go8p	1kapp	55.8
1dzba	1qkzh	55.8
1du5b	1rqwa	55.8
1tg6b	1tyff	55.7
1ny72	1bmv2	55.7
1a65a	1v10a	55.7
1vbga	2dika	55.4
1gv3b	1mngb	55.4
1aoka	1tjka	55.4
2dquh	2mpah	55.3
1yd8h	1x79a	55.3
1uhua	1mn8a	55.3
1gdva	1kibh	55.3
2pvba	1rwyc	55.2
1scth	1hbia	55.2
1o6sb	1ncia	55.2
1ewxa	1oc9a	55.2
1vl2d	1kord	55.1
1tw6a	1nw9a	55.1
1sm4b	1b2ra	55.1
1ufrd	1a4xb	54.9
1xu4a	1pzng	54.8
1aokb	1tgma	54.5
1qlrc	1nakm	54.3
1c5fg	1cyna	54.3
1o6cb	1f6da	54.2
1kuga	2aigp	54.1
1qwma	1qqwd	54.0
1f58h	1axth	54.0
1si8d	8cata	53.9
1ls6a	1xv1b	53.8
2rmcg	1qnga	53.7
1z68b	1orwd	53.7
1tzgm	1ct8c	53.6
1wnrg	1hx5a	53.5
1qxhb	1xvqa	53.5
1s7ya	1ftja	53.4
1viwa	1xd1a	53.3
1z4ra	1pu9a	53.2
1nexc	1fqvb	53.2
1e0va	1fh9a	53.2
1stfi	1gd3a	53.1
1lrka	1hzjb	53.1

1upnb	1rhi2	52.8
1svzb	1tjgl	52.8
1oi7a	1euda	52.8
1ub0a	1jxhb	52.7
1c5ia	1te1b	52.6
1j2pa	1pmao	52.5
1huna	1u4mb	52.5
1buvm	1jiza	52.5
1on9d	1xnwf	52.4
1if1a	2irfl	52.4
1mvma	1c8ea	52.3
1v5ba	1osid	52.2
1h5yb	1gpwa	52.2
2vitb	1b4jh	52.1
1o68b	1m3ua	52.1
1yv7a	1oj8a	52.0
1yedb	1dbbh	52.0
1b98a	1sgfy	52.0
1b2pa	1nivc	51.9
2sebd	1b1za	51.8
1nuea	1s59b	51.7
3crol	1perl	51.6
1tzgi	2hmid	51.6
1mhzg	1fyze	51.5
1mhwa	1s4vb	51.5
1wm3a	1y8rf	51.4
1u9kb	1smob	51.4
1r0ob	1dsza	51.4
1ehwb	1b4sa	51.4
1dfbh	1faih	51.4
1jj4a	1r8he	51.2
1fbiq	1jnhf	51.2
1e9ib	1oepa	51.2
1xq9b	1riib	51.1
1t9kd	1t5oa	51.1
1r5qa	1q6bb	51.1
1quaa	1nd1a	51.0
1k5jd	1xe0c	51.0
1c5cl	1sbsl	51.0
1itza	1r9jb	50.9
1f3rb	1q1ji	50.8
1bj3a	1jwia	50.8
1dzqb	1wblb	50.7
1cxvb	1hv5a	50.7
1wwbx	1wwwy	50.6
1rpna	1t2ad	50.5
2eifa	1iz6b	50.4
1mq0b	1jtka	50.4
1a6vh	1fvcd	50.4
1d4m3	1vrh3	50.2
1br2b	1kk7a	50.2
1tejb	1j2la	50.0
1soxb	1ogpe	50.0
1qqya	1dzbx	50.0
1kj1p	1b2pb	50.0
1bn6a	1mj5a	50.0
1zfja	1eepb	49.9
1ygpb	1z6pa	49.9

1y18e	1r24c	49.8
1tzgh	1ai1h	49.8
1r1a3	1ev13	49.8
1vkoa	1jkfa	49.7
1oc2b	1bxka	49.7
1nb2a	1w7wa	49.7
1a78a	1sltb	49.6
5mdhb	1b8ua	49.5
1tmha	1spqa	49.4
1oria	1g6q1	49.4
1dc6a	1obfp	49.4
1qagb	1dxxa	49.3
1xrga	1qahb	49.2
1s03g	1seib	49.2
1pp2l	1oqsh	49.2
4gpd2	1vc2a	49.1
1k3ca	1xkva	49.0
1gv7a	2rlne	49.0
1epxd	1qo5r	48.9
1vlgh	1s3qe	48.8
1f28b	1aiqa	48.8
1v9u3	1upnc	48.7
1if2a	1mo0b	48.6
1q4ra	1tr0y	48.5
1n9la	1jnud	48.5
1hcqf	1r0nb	48.5
1bqba	1kroa	48.5
1b8ra	1ttxa	48.5
1fp1d	1kyze	48.4
1uqre	1d0ia	48.3
1do5b	1srdd	48.3
1kq3a	1jpua	48.2
1hwue	1vfja	48.1
1ex6b	1lvga	48.0
1we3g	1ioka	47.9
1t8pb	1bq3d	47.8
1h4ga	1xypb	47.8
1fbna	1nt2a	47.6
1mjgb	1jqka	47.5
1o0ia	1vh9b	47.4
1jb9a	1ewyb	47.4
1a6vj	1dl7h	47.3
1spgb	1yffh	47.2
1jsma	1jsda	47.2
1i6qa	1qjma	47.2
1aeia	1yj0a	47.2
1sb3c	1n63d	47.1
1o60d	1fxpb	47.1
1eg1c	2a39a	47.1
1dfob	1bj4a	47.1
1rlub	1w5eh	46.9
1uz1a	1uyqa	46.7
1mula	1p78b	46.7
1kent	1yuhb	46.6
1k6aa	1isva	46.6
1jazb	3pga1	46.5
1umcd	1dtwb	46.4
1rmva	1vtmp	46.4

1we1d	1s8cd	46.3
1b0xa	1f0ma	46.3
1w3ua	1bjna	46.2
1qs0b	1w88f	46.2
1b3ba	1nr1b	46.2
1qzta	1xcof	46.1
1ui9a	1coma	46.0
1qu9a	1onia	46.0
1jpth	1ghfh	45.9
1gu6g	1qdba	45.9
1nqbc	1lo4h	45.8
1gyqa	1rm3o	45.8
1czha	1ag9a	45.8
1uata	1etjd	45.7
1ep7a	1v98a	45.7
1buda	1atla	45.7
1nmla	1iqcd	45.6
1apya	2gawc	45.6
1s1ib	1s72a	45.5
1pwba	1bv4a	45.5
2cgrh	1nj9b	45.4
1v8fa	1n2jb	45.4
1rscm	1ir1s	45.4
1oaqh	2dlfh	45.4
1m3va	1j2oa	45.4
1p2qa	1h9ie	45.2
1h95a	1g6pa	45.2
1umvx	1aypb	45.1
13pka	1fw8a	45.1
1nxja	1j3la	45.0
1g0ca	1w3ka	44.8
1uzfa	1j38b	44.7
1fnza	1v6oa	44.7
43cab	1jtpa	44.6
1iueb	1frrb	44.6
1eyla	1ba7b	44.6
1vh1d	1gq9a	44.4
1iisc	1j86b	44.4
1gw5s	1w63x	44.4
1whja	1whha	44.3
1i8fe	1h64c	44.3
1e17a	1d5va	44.3
1h1ya	1tqjf	44.1
4vgcb	1uhba	44.0
1v5wb	2bkea	44.0
1c5ch	1cloh	44.0
1xdfb	1b6fa	43.9
1qn2b	2pcbb	43.9
1p4sa	2eckb	43.9
1n86b	1m1jf	43.9
1lgab	1ly8b	43.9
1dlkb	1tawa	43.9
1m9wa	1b3ia	43.8
1v2aa	1jlva	43.7
1s0ha	1vwtd	43.7
1lwue	1jc9a	43.7
1gqon	2dhqa	43.7
1x94b	1tk9a	43.6

1uptc	1r8qb	43.6
1uj1b	1lvoa	43.6
1l2ea	1h4wa	43.6
1i8ta	1v0jd	43.6
1ahjc	1ugra	43.6
1ulub	1jw7d	43.5
1qfwh	1rjca	43.4
1b0aa	1a4ia	43.4
1k44b	1xiqb	43.3
1v9na	1rfmh	43.2
1sv0a	1lkye	43.2
1dt2a	1agja	43.2
1aj9a	2hhed	43.2
1rhi1	1upna	43.1
1qcfa	1k9aa	43.1
1oxhb	1w0ib	43.1
1os6a	1lm2a	43.1
1jjxa	2cbsa	43.1
1r6na	1tueg	43.0
1kyoj	1kxqh	43.0
1ezra	2masd	43.0
1w2va	1xyfb	42.9
1udea	2eipa	42.9
1st7a	1nvla	42.9
1g0wa	1p52a	42.9
1egna	3ovwb	42.9
1c7ma	1io3a	42.9
1buia	3btge	42.9
1fzdh	1n73c	42.8
1vgwc	1h3ma	42.7
1v9aa	1s4da	42.6
1jm4b	1e6ia	42.6
1qapb	1qprf	42.5
1djub	1b5oa	42.5
2bbva	1f8va	42.4
1nbuc	1sqlp	42.4
1s72i	1hc8a	42.3
1f2ed	2pmtd	42.3
1azyb	1brwb	42.3
1y7ub	1vpma	42.2
1tzaa	1xq4d	42.2
1okbb	1udie	42.2
1nj1a	1h4qa	42.2
1mema	1m6db	42.2
1fiqa	1ffvd	42.2
1qf6a	1nyra	42.1
1iwea	1adea	42.1
1o6ba	1b6ta	42.0
1jb5b	1gy7b	42.0
1agwb	1fvra	42.0
1y6pb	1m8sa	41.9
1s1it	1s72s	41.9
1mzhb	1j2wa	41.9
1iqdc	1czsa	41.9
1fatd	1bzwd	41.9
1aym1	1hri1	41.9
1mioc	1fp4a	41.8
1lgba	1v6la	41.8

1k5ca	1czfa	41.8
1b9bb	1m6jb	41.8
1g09c	1g09d	41.7
4prch	1l9jt	41.5
1yl3n	1jj2j	41.5
1uv4a	1gyhe	41.5
1szbb	1nzib	41.5
1n4oa	2cc1a	41.5
1mjtb	1nsia	41.5
1fi8b	1fujd	41.5
1uzya	1v6id	41.4
1p7gh	1d5na	41.4
1p57b	1v2qt	41.4
1dt0a	1gn4d	41.4
1ifvb	1e09a	41.3
1ci0a	1jnwa	41.2
1ad1a	1twsb	41.2
1rz2a	1ng5a	41.1
1q4qa	1xb0a	41.1
1azsc	1shzd	41.1
1w0dd	1cnzb	41.0
1sdxa	1ieja	41.0
1qfkh	1v2rt	41.0
1o8ba	1uj4a	41.0
1mp9b	1cdwa	41.0
1m1ma	1ub7d	41.0
1jswc	1fuoa	41.0
1glia	1fhjd	41.0
1fdhg	1dxva	41.0
1fcja	1m54f	41.0
1bjfb	1s1ea	41.0
1wueb	1r0mb	40.9
1jd5a	1oy7e	40.9
1j7jb	1p17c	40.9
1f0pa	1r88b	40.9
1w74b	1istb	40.8
1v8aa	1c3qa	40.8
1r4ra	2nllb	40.8
1evqa	1jjid	40.8
1pklb	1pkyc	40.6
1n8yc	1nqla	40.6
1icqa	1bwla	40.6
1g82b	1k5va	40.6
1b4ea	1b4kb	40.6
1rqqa	1m52b	40.5
1g82d	1k5vb	40.5
5p2pa	1xxsb	40.4
1g2xa	1gmza	40.4
1bpwd	1bxsd	40.4
1yx2b	1vloa	40.3
1vbjb	1k8ca	40.3
1nfgd	1gkpa	40.3
1n47d	1cq9d	40.3
1ml1c	1lyxa	40.3
1cc1l	1ubtl	40.3
1cbra	2ansb	40.3
1pobb	1a2ah	40.2
1n0ra	1blxb	40.2

1gmma	1w9sa	40.2
1s1pa	1hqta	40.1
1ma1b	1qnnd	40.1
1wdfa	1wncc	40.0
1m1nb	1miod	40.0
1f6rd	1iora	40.0
1ksha	1z6xb	39.9
1frfs	1cc1s	39.9
1bqya	1a0jd	39.9
3rpba	1uowa	39.7
1uh7a	1pple	39.7
1o5ob	1bd3d	39.7
1v4uc	1outb	39.6
1uaxb	1ekea	39.6
1jbaa	1g8ib	39.6
1hfob	1mfia	39.6
1tc8a	1b4wb	39.5
1sk3a	1ohta	39.5
1ki9a	1nksf	39.5
1qlea	1ffta	39.4
1j15a	1xx9b	39.4
1t47b	1sqda	39.3
1q3ta	1kdtb	39.3
1ggpb	1m2tb	39.3
2btma	7tima	39.2
1v6hc	1vhfa	39.2
1k1dh	1kcxb	39.2
7aata	1czea	39.1
3tatb	1ay4b	39.1
1xmrb	1w4ra	39.1
1vyca	1hc9a	39.1
1p73c	1e2ha	39.1
1dlya	1uvya	39.1
1c40a	1a9wf	39.1
1buna	1aypc	39.1
1va4b	1brob	39.0
1u79c	1a7xa	39.0
1oe9a	1yv3a	39.0
1azya	1uoua	39.0
2tsra	1bkoa	38.9
1wndd	1of7h	38.9
1r52c	1umfd	38.9
1oeca	1byga	38.9
1gh1a	1siya	38.9
1ylva	1w5qb	38.8
1my6b	1ap5a	38.8
2bg9a	2bg9e	38.7
1v9u1	1bev1	38.7
1t3ba	1tjda	38.7
1q45b	1vyra	38.7
1u00a	1dkxa	38.6
1gnza	1v6ja	38.6
1adjb	1kmnc	38.6
1uvpa	1v2pt	38.5
1j3ca	1qrvb	38.5
1biia	1de4a	38.4
1wy1a	1rtyb	38.3
1uvth	1zzza	38.3

1spga	1nqpd	38.3
1nkpd	1an2a	38.3
1rx0c	1ukwb	38.2
1nyud	2bhka	38.2
1jqib	1t9gd	38.2
1y6hb	1xeoa	38.1
1tcoa	1fjma	38.1
1rv0h	2visc	38.1
1pffb	1cs1d	38.1
1m8tb	1vapb	38.1
1hzob	1lhya	38.1
1gdua	1avwa	38.1
1pcia	1by8a	38.0
1gehc	1svda	38.0
1a8jh	1nsnl	38.0
1txja	1h6qa	37.9
1sdma	1yrsa	37.9
1p7qd	1p6fa	37.9
1kz9a	1nqub	37.9
1vj1a	1v3ta	37.8
1owga	1b8zb	37.8
1mqbb	1k3aa	37.8
1g2oa	1vmkb	37.8
1ulqc	1pxtb	37.7
1s95b	1m63a	37.7
1pfxl	1w0yl	37.7
1nwgc	1di3a	37.7
1m2ob	2j5xa	37.7
1kijb	1s16b	37.7
1jgka	1rgja	37.7
1jagh	1j90a	37.7
1gera	1feca	37.7
1yaad	1cq8a	37.6
1i3od	1qdub	37.6
1apxd	1u75c	37.6
1ajrb	1togb	37.6
1szpd	1pzna	37.5
1n7ka	1ub3d	37.5
1cmza	1fqia	37.5
1re0b	1r8me	37.4
1ijta	1axmd	37.4
1f56b	1ws8d	37.4
1dzra	1upia	37.4
1c41f	1rvv4	37.4
1vmja	1ve0a	37.3
1n8pd	1gc0c	37.3
1rj5a	1j9wb	37.2
1mbqa	1jbuh	37.2
1kbvd	1rzqa	37.2
1pfxc	2a45e	37.1
1i8oa	1fi9a	37.1
1f82a	1t3ab	37.1
1dsya	1k5wa	37.1
2notb	1jlta	37.0
1g292	1oxsc	37.0
1d9ke	1oaym	37.0
1c41g	1w19a	37.0
1xw9a	1fb0b	36.9

1sxjd	1sxjb	36.9
1eumc	1s3qd	36.9
1y4ta	1si1a	36.8
1owsb	1aypa	36.8
1n1ab	1u79d	36.8
1m2ta	1il5a	36.8
1k38a	1h5xa	36.8
1jgxm	1dxrl	36.8
1pyoc	1bmqa	36.7
1g291	1oxvd	36.7
2hpqp	1tpkc	36.6
1qmvf	2cv4f	36.6
1nfdh	1aj7h	36.6
1n6mb	1goja	36.6
1k79a	1puef	36.6
1gpzb	1d9ia	36.6
1l1ra	1g2pa	36.5
1gbra	1udla	36.5
1ybwa	1gvla	36.4
1sk7a	1p3va	36.4
1ovma	1qpbb	36.4
1lulb	1cr7a	36.4
1jzaa	1fh3a	36.4
1vp2b	1b78a	36.3
1qqka	1k5ub	36.2
2mlla	1hwma	36.1
1nr9b	1nkqa	36.1
1epza	1pm7a	36.1
1viwb	1s1aa	36.0
1v4wb	1outa	36.0
1s1il	1s72m	36.0
1iq7a	1h43a	36.0
1ie0a	1joea	36.0
1hwoa	1rzoc	36.0
1bawa	1id2a	36.0
1xi9b	1gckb	35.9
1wxdb	1nvtb	35.9
3sgae	1boqa	35.8
1xfja	1rv9a	35.8
1vftb	1bd0a	35.8
1ti3a	1m7ta	35.8
1kw4a	1pk1d	35.8
1hgxa	1p19b	35.8
1brup	1hj8a	35.8
1yn4a	1yn3b	35.7
1tme2	1eah2	35.7
1p15a	1bzha	35.7
1iq9a	1lxha	35.7
1autl	1xkal	35.7
1hlqa	1ckub	35.6
1f7sa	1q8xa	35.6
1e57a	1auya	35.6
2bmib	1dd6b	35.5
2bklb	1e5ta	35.5
1wuub	1s4ed	35.5
1snya	1yo6a	35.5
1n9wa	1asya	35.5
1j8mf	1ls1a	35.4

1gkna	1ghqc	35.4
2mev2	1bev2	35.3
1smaa	1izja	35.3
1r6xa	1v47a	35.3
1na8b	1gyua	35.3
1j99a	1aqua	35.3
1hv8b	1t6nb	35.3
1fpra	1p15b	35.3
1elka	1lf8d	35.3
1dy6b	1htzf	35.3
1p2ta	1xtra	35.2
1m3sb	1vimd	35.2
1qbmh	1q72h	35.1
1m3ga	1vhrb	35.1
1f5jb	1t6gd	35.1
1s1im	1k8ak	35.0
1nena	1qlbd	35.0
1yfob	1wcha	34.9
1t3qf	1n5wf	34.9
1hiza	1ta3b	34.9
1w96c	1dv2a	34.8
1pyoa	1i51c	34.8
1elva	1autc	34.8
1efpb	1o96e	34.8
4ts1a	1y42x	34.7
1vrda	1nf7b	34.7
1s1gb	1t1da	34.7
1mj4a	1do9a	34.7
3znba	1mqoa	34.6
1vg8a	1u90b	34.6
1so2c	1y2kb	34.6
1hzdb	1duba	34.6
1a5hb	1p2ma	34.6
3btaa	1epwa	34.5
1cdtb	1mr6a	34.5
1w7oa	2ctha	34.4
1onfa	1typa	34.4
1kyof	1be3h	34.4
1a25a	1w15a	34.4
1z82b	1evya	34.3
1v9lb	1nr7l	34.3
1uv0a	1fvua	34.3
1t8ha	1rw0b	34.3
1nxkb	1ql6a	34.3
1gkga	1ly2a	34.3
1dofd	1c3ca	34.3
1xrha	1rfmg	34.2
1hzfa	1qsjd	34.2
1f6wa	1mx1d	34.2
1g0ua	1g65d	34.1
1fi8a	1spja	34.1
1f4oa	1u5ib	34.1
1bzya	1r3ub	34.1
1t0oa	1r46b	34.0
1qxwa	1c21a	34.0
1qpca	1r0pa	34.0
1kz9c	1c2ym	34.0
1ivhd	3mdda	34.0

1duvg	1yh0a	34.0
3pcdn	1eoaa	33.9
1rjxb	1ycph	33.9
1ob9a	1gefa	33.9
1kb0a	1w6sc	33.9
1eyvb	1tzxb	33.9
1c1la	1qmjb	33.9
1xbfc	1vphf	33.8
1osdb	1p8ga	33.8
1lwia	1mzrb	33.8
1jtzx	1d0ga	33.8
1iw0b	1dvgb	33.8
2hgsa	1m0tb	33.7
1u2ea	1ukba	33.7
1nxca	1hcuc	33.7
1r9wa	1ksxa	33.6
1mt0a	1l2tb	33.6
1avbb	1v6ma	33.6
1v3ya	1rl4a	33.5
1ng0a	1f2na	33.5
1mamh	2f5ah	33.5
1avba	1v6od	33.5
1wqla	1eg9a	33.4
1jmjb	1hlea	33.4
3raps	1tx4b	33.3
2fgwh	1a5fh	33.3
1xkna	2cmua	33.3
1xbca	1fvrb	33.3
1vffa	1pbgb	33.3
1uexb	1jzna	33.3
1uema	1ueya	33.3
1ts9a	1v76b	33.3
1tkub	1pvwb	33.3
1owsa	1pc9b	33.3
1oscf	1j2va	33.3
1nfha	1udvb	33.3
1lcya	1te0b	33.3
1l9na	1evua	33.3
1j30a	1s30a	33.3
1b1ya	1j18a	33.3
1qqp2	1mec2	33.2
1n60e	1t3qe	33.2
1jpaa	1u4db	33.2
1ibjc	1qgnh	33.2
1flga	1g72c	33.2
1fiza	1fxya	33.2
1bjre	1ea7a	33.2
1s72h	1s1ii	33.1
1xmcb	1ndba	33.0
1ugpb	1ahjb	33.0
1f20a	1ddga	33.0
3sxla	1cvja	32.9
1znca	1v9ea	32.9
1yoya	1xx7c	32.9
1xkka	1lufa	32.9
1ry6a	1mkja	32.9
1qfna	1b4qa	32.9
1j5ka	1dt4a	32.9

1c7ub	1mnma	32.9
1qpxb	1quni	32.8
1pvwa	1k4pa	32.8
1mhma	1jl0a	32.8
1geqb	1a5ba	32.8
1bola	1dixa	32.8
1yh1a	1vlva	32.7
1urzc	1okeb	32.7
1q6ga	1b90a	32.7
1pdxa	1ayfa	32.7
1np7b	1iqua	32.7
1mhcd	1zagd	32.7
1lm4a	1rl4b	32.7
1h6vb	2tprb	32.7
1bwvy	1wddw	32.7
1py1c	1elkb	32.6
1xj5a	1inla	32.5
1sefa	1sfnb	32.5
1q1ua	1iila	32.5
1n7fb	1v5qa	32.5
1klka	1ddra	32.5
1bxva	1t5kd	32.5
1v2xa	1gz0a	32.4
1s1hb	1vi6c	32.4
1qpda	1r1wa	32.4
1p1aa	1aara	32.4
1geqa	1k7ea	32.4
2lefa	1i11a	32.3
1uulf	1yepe	32.3
1eu5a	1q5hb	32.3
1blba	1m8ua	32.3
1qdqa	1ms6a	32.2
1lbla	1i4nb	32.2
1jgca	1nfvp	32.2
1jbbb	1kpsc	32.2
1yhuh	1yhuf	32.1
1u9ca	1qvvd	32.1
1tqyg	1dd8d	32.1
1svcp	1nfic	32.1
1la6b	1qpwc	32.1
1gvza	1bqyb	32.1
1gsub	1tu7b	32.1
1fssb	1je9a	32.1
1uhwa	1v3fa	32.0
1q1la	1sq1a	32.0
1kxtb	1dqll	32.0
1i2wb	1g6aa	32.0
1gz6d	1i01c	32.0
1eiia	2cbra	32.0
1dwfm	1ug6a	32.0
1dica	1o5fh	32.0
1agre	1dk8a	32.0
2cv4c	1e2yg	31.9
1z2ua	1yh6b	31.9
1xwya	1yixb	31.9
1pfzc	1flha	31.9
1mg8a	1sifa	31.9
1i6ua	1yl4k	31.9

1ggpa	1d8va	31.9
4caaa	1atta	31.8
1ur1a	1w3hb	31.8
1rmfh	1otse	31.8
1m79a	1drab	31.8
1fiwa	1bdaa	31.8
2cxba	1i55b	31.7
1yxmc	1geef	31.7
1v27a	1byna	31.7
1eiga	1hfna	31.7
1rqga	1f4la	31.6
1pwod	1mg6a	31.6
1m78b	1ddsb	31.6
1eaza	1u2ba	31.6
1dv8a	1c3ab	31.6
2a2ud	1a3ya	31.5
1t3mb	1apzd	31.5
1q5qi	1pmat	31.5
1pwaa	1ry7a	31.5
1p9du	1q0wb	31.5
1nn4b	1usle	31.5
1mvfb	1ao7d	31.5
1k2aa	2rnfb	31.5
1dc2a	1svxa	31.5
1wcua	1gwka	31.4
1vjoa	1iugb	31.4
1uqza	1xyzb	31.4
1spva	1hjza	31.4
1g0ha	1lbzb	31.4
1d7oa	1qsgh	31.4
1wmsa	2ngra	31.3
1qe3a	1qo9a	31.3
1pytc	1fdpb	31.3
1lgyc	1dt3a	31.3
1kgzb	1gxba	31.3
1xu0a	1e1ja	31.2
1uxaa	1h7zb	31.2
1ulna	1ehda	31.2
1muqa	1v7pa	31.2
1hncb	17gsa	31.2
1gcwc	1gbud	31.2
1a3fb	1s8ga	31.2
1yl3q	1ilya	31.1
1vbwa	1meei	31.1
1rdua	1t3va	31.1
1orrc	1kewb	31.1
1mhpb	1idn2	31.1
1ky2a	1xtsa	31.1
1kk3a	1exma	31.1
1i3of	1qbha	31.1
5ruba	1iwaa	31.0
1vlpb	1yird	31.0
1s1hg	1yl4j	31.0
1qava	1ujda	31.0
1ncbn	1vcja	31.0
1m2pa	1h08a	31.0
1lpja	1vyga	31.0
1gq5a	1whaa	31.0

1z8qa	1f4ta	30.9
1t9sa	1zknd	30.9
1mjfa	1iy9d	30.9
1h32b	1hrob	30.9
1e12a	1uazb	30.9
1ae1b	1ahia	30.9
4prcm	1aigl	30.8
1v9tb	1yndb	30.8
1azzb	1rfna	30.8
1w9pb	1owqa	30.7
1v2eb	1o4sb	30.7
1u2xb	1l2la	30.7
1rzrg	1bdha	30.7
1ns5b	1vh0b	30.7
1ns5a	1vh0a	30.7
1ixec	1owcb	30.7
1itxa	1e9la	30.7
1howa	1w98a	30.7
1f2va	1ou0b	30.7
1eufa	1nrnh	30.7
1xsna	2bpfa	30.6
1qmna	1c8oa	30.6
1lmha	1bs4a	30.6
1am9b	1hloa	30.6
1xg2a	1qjvb	30.5
1ri8a	1pw3b	30.5
1oo8a	1oyhl	30.5
1mec3	1nd2c	30.5
1iy8h	1ydeb	30.5
1fntf	1g0ub	30.5
1bmaa	1f5la	30.5
1yh6a	1i7kb	30.4
1xjsa	1wfza	30.4
1v1ga	1bjfa	30.4
1qada	1ijra	30.4
1pytd	1au8a	30.4
1ppge	1xxfa	30.4
1kfwa	1lg2a	30.4
1hkwb	1twid	30.4
1gukb	1pa3a	30.4
1cyda	1xg5b	30.4
1adwa	1tu2a	30.4
1un7b	1ymya	30.3
1qyab	1xuab	30.3
1iruz	1q5qh	30.3
1gz6c	1g6kf	30.3
1c8nb	1vb4a	30.3
1ursb	1mdp2	30.2
1snzb	1mn0b	30.2
1q0ub	1qdea	30.2
1o91a	1pk6c	30.2
1ldnh	1uxja	30.2
1jyua	1csza	30.2
1x9da	1krfb	30.1
1s6ua	1y3ka	30.1
1olxa	1w85a	30.1
1m06g	1gff2	30.1
1jwoa	1oo4a	30.1

1f3aa	1baya	30.1
1x9fb	1yhug	30.0
1rjwd	1e3ib	30.0
1r8pb	1jjhc	30.0
1pz1b	1lqaa	30.0
1ozsa	1j7oa	30.0
1muoa	1ydre	30.0
1m8ab	1esra	30.0
1m07a	1z3pe	30.0
1jgja	1pxsb	30.0
1ijca	1w6ba	30.0
1i10a	1sowa	30.0
1ctpe	1p4fa	30.0
1bvub	1hwxa	30.0
2bmcf	1v0oa	29.9
2bija	1l8ka	29.9
1se6a	1gwib	29.9
1nfba	1mewa	29.9
1gz7d	1b41a	29.9
1fuea	1xt6a	29.9
1ar63	2mev3	29.9
1ylob	1y0ya	29.8
1yb4b	2cvzd	29.8
1nshb	1mr8b	29.8
1m9ua	1ltod	29.8
1kigh	1k2i1	29.8
1evha	1i7ad	29.8
1dk5b	1mcxa	29.8
1z33a	1t0ub	29.7
1ycna	1g5na	29.7
1uweh	1nl0l	29.7
1h7za	1p6aa	29.7
1euhb	1uzbb	29.7
1euha	1a4sa	29.7
1ebba	1c7za	29.7
1uwtb	1hxja	29.6
1smla	1k07a	29.6
1xq5c	1cg5b	29.5
1nn6a	1eawa	29.5
1mc7a	1ujua	29.5
1rd5a	1wq5b	29.4
1n4ha	1exaa	29.4
1mvea	1ajka	29.4
1mdja	1v98b	29.4
1ji4e	1umni	29.4
1gulc	10gsa	29.4
1g9oa	1wfva	29.4
1g65v	1pma2	29.4
1ef7a	1nb5c	29.4
1bypa	1mdab	29.4
1rqqc	1i3za	29.3
1i0zb	1t2da	29.3
1g5ub	1d1jc	29.3
1d5ca	1i2mc	29.3
1knra	1e7pa	29.2
1f6fb	1a22b	29.2
1bkza	1hlcb	29.2
5ukda	1zakb	29.1

1y5ea	1jljc	29.1
1xfla	1xoba	29.1
1vq3d	1t4aa	29.1
1uzmb	1hxhd	29.1
1lg9a	1egya	29.1
1k3bb	1khpa	29.1
1iruo	1g65c	29.1
1i1na	1vbfd	29.1
1gtub	22gsb	29.1
1ep1a	1ovdb	29.1
1bvld	1ktrh	29.1
2h1ph	1egjh	29.0
1uc3d	1a4fa	29.0
1sdja	1xuba	29.0
1rbaa	1uppg	29.0
1pxfa	1pybd	29.0
1by7a	1b3ka	29.0
1w9xa	1mwoa	28.9
1ukcb	1p0pa	28.9
1q17c	1yc2d	28.9
1ls5b	1avfj	28.9
1fbvc	1kpsa	28.9
1dztb	1nzcd	28.9
1uiya	1sg4a	28.8
1sumb	1t72a	28.8
1sgfz	1b0fa	28.8
1qoua	1bd9b	28.8
1pzsa	3sodo	28.8
1qd9a	1pf5a	28.7
1jy5a	1j1fa	28.7
1f9mb	1rqma	28.7
1qdda	1oz7a	28.6
1iodb	1fm5a	28.6
1ie3d	1uxka	28.6
1f2la	1b53b	28.6
1e3sa	2bgma	28.6
1bzqn	1qp1c	28.6
1b3oa	1me8a	28.6
1ev4c	1tw9b	28.5
1chrb	1tkkh	28.5
6gsub	14gsb	28.4
2bmcc	1ua2d	28.4
1yspa	1td5b	28.4
1wu2b	1t3eb	28.4
1sxtb	1et9a	28.4
1p27d	1no8a	28.4
1o5id	1rwba	28.4
1mw4a	1ju5a	28.4
1kona	1mw7a	28.4
1kj2a	1xfpa	28.4
1iq3a	1c07a	28.4
1hnee	1npmb	28.4
1h6ky	1rk8a	28.4
1h6kx	1oo0b	28.4
1gumh	17gsb	28.4
1apme	1nvqa	28.4
1ujpa	1a50a	28.3
1uf9a	1t3hb	28.3

1uexa	1k9jb	28.3
1sgfa	2bm2a	28.3
1jy5b	1iqqa	28.3
1b4pa	1px6b	28.3
1whga	1whla	28.2
1wg6a	1um1a	28.2
1qx2b	1mq1a	28.2
1mzda	1eq9b	28.2
1lurb	1l7ja	28.2
1j71a	1czie	28.2
1ff4a	1abta	28.2
1f2ri	1ibxa	28.2
1dqlh	2cd0b	28.2
1cg7a	1wgfa	28.2
1ae1a	1doha	28.2
1ku5b	1p3mb	28.1
1i01f	1pr9b	28.1
1hrdc	1l1fb	28.1
1gwia	1uedb	28.1
2u2fa	1hd0a	28.0
1pk6a	1c28a	28.0
1liak	1kn1b	28.0
1hbka	1hb8c	28.0
1g16d	1ds6a	28.0
1g0va	1oexa	28.0
1ayza	1y8xa	28.0
2duba	1q51a	27.9
1pdub	1p8da	27.9
1oaja	1ozua	27.9
1jebc	1myib	27.9
1dsfh	1igml	27.9
1cpza	1kqka	27.9
1xe3a	1t0ua	27.8
1rusa	1rldb	27.8
1ojua	1pzea	27.8
1m31b	1irjd	27.8
1fjca	1h2vz	27.8
1f99a	1b33c	27.8
1bzyb	1pzma	27.8
1u8xx	1up7g	27.7
1fyra	1luna	27.7
1c4zd	1q34a	27.7
1xjda	1lewa	27.6
1uu9a	1oguc	27.6
1ueqa	1uhpa	27.6
1k2wb	1gcof	27.6
1k0ka	1hlup	27.6
1dvva	1w2la	27.6
1cydd	1mxfa	27.6
1bbt3	1rhi3	27.6
1b0ua	1g6ha	27.6
1a0qh	2ckbb	27.6
2nlra	1ks5a	27.5
1vcka	1sjga	27.5
1nhza	1qkna	27.5
1b34b	1i4kh	27.5
1b00a	1udra	27.5
1ysqa	1td5a	27.4

1wepa	1wesa	27.4
1ujya	1u5sa	27.4
1prxb	1qmvd	27.4
1ie5a	1g1cb	27.4
1wa3d	1mxsa	27.3
1s4qa	1z6ga	27.3
1qk3c	1hgxb	27.3
1qf9a	1s3ga	27.3
1hg4a	3erdb	27.3
1pzva	1yh2a	27.2
1m2xc	1ko3a	27.2
1ia8a	1q5kb	27.2
1ezva	2bcca	27.2
1dc9a	1o1va	27.2
1ukia	1z57a	27.1
1rzxa	1q3pb	27.1
1p93a	1ndea	27.1
1mqvb	1rcpb	27.1
1hq8a	1v4lb	27.1
1fi5a	1avsa	27.1
1cjxa	1sp8c	27.1
1bila	2rmpa	27.1
1v8ja	1ia0k	27.0
1n1qc	1uvha	27.0
1h5ba	1ivlb	27.0
1h0ba	1uu5a	27.0
1cpjb	1o0eb	27.0
1c5ga	1attb	27.0
1b9ra	1i7ha	27.0
5lvea	1mvfa	26.9
1ujva	1ihjb	26.9
1tw4b	1opaa	26.9
1qpwd	1jw8a	26.9
1qfwm	1ohqa	26.9
1e83a	1a7va	26.9
1szsd	1dtyb	26.8
1ouwa	1ws4g	26.8
1ovlc	1fd0a	26.7
1iu2a	1n7ea	26.7
1h3ph	1qsed	26.7
1ydmb	1u3ga	26.6
1xkba	1hj7a	26.6
1w7la	1j32b	26.6
1qgc2	1mqtb	26.6
1prxa	1qmvj	26.6
1ioob	1bk7a	26.6
1wjla	1um7a	26.5
1ty0b	1bxtb	26.5
1s9ja	1v0ba	26.5
1s1ig	1s72f	26.5
1k9ih	1yttb	26.5
1ef7b	1cqdd	26.5
2plda	1a1aa	26.4
1xz0c	1bd2b	26.4
1urha	1h4mx	26.4
1u2ed	1j1ia	26.4
1skxa	1uhlb	26.4
1pafb	1bryz	26.4

1mx7a	1ftpb	26.4
1hqva	1aj5a	26.4
1peyb	1jbea	26.3
1g9ea	1qnzl	26.3
1ebdb	1tytb	26.3
1rvfl	1ar1c	26.2
1q44a	1g3mb	26.2
1pr1a	1ryzd	26.2
1nytd	1npda	26.2
1n7ta	1n7fa	26.2
1meha	1jcnb	26.2
1jboa	1ha7h	26.2
1ha7g	1gh0x	26.2
1cjxd	1sp9a	26.2
1pr5a	1k3fa	26.1
1oeda	1oede	26.1
1mvna	1g63f	26.1
1k66b	1krwa	26.1
1hr8b	2bccb	26.1
1f3md	1jowb	26.1
1dupa	1f7ra	26.1
1ue8a	1pkfa	26.0
1ty6h	1mj8h	26.0
1rpyb	1rjaa	26.0
1itoa	1u9qx	26.0
1awcb	1mx6a	26.0
1xvpd	1nava	25.9
1vhkb	1nxzb	25.9
1qdla	1k0ga	25.9
1nytc	1npdb	25.9
1ot8c	1ixva	25.8
1ir0a	1sj1b	25.8
1egga	1b08c	25.8
1opza	1s6oa	25.7
1nnqb	1b71a	25.7
1kxvd	1bwwa	25.7
1h0bb	1olqb	25.7
1fod3	1bev3	25.7
1eeqb	1op9a	25.7
1brea	1qfwi	25.7
3psra	1odbf	25.6
1xq1a	1g0na	25.6
1o8pa	1uz0a	25.6
1bw9b	1v9lc	25.6
1sc4a	1f9ea	25.5
1nzyb	1rjmb	25.5
1ntoa	1ee2b	25.5
1e92c	1vl8b	25.5
1udrb	1srrc	25.4
1s6ia	1lkja	25.4
1rh8a	1ugka	25.4
1pt6b	1rd4d	25.4
1jkga	1qmad	25.4
1f51e	1a0oa	25.4
1f3bb	4gtuh	25.4
1at3b	1cmva	25.4
5tmpa	1g3ua	25.3
4tmka	1w2hb	25.3

1uepa	1mfla	25.3
1k8ia	1d6ea	25.3
1telb	1wdde	25.2
1smkd	1hyea	25.2
1igmh	1ap2a	25.2
1ulua	1spxa	25.1
1rjka	2prgb	25.1
1ogae	1fpth	25.1
1w63c	1gw5a	25.0
1v7pb	1wmyb	25.0
1ukvy	1doaa	25.0
1nama	1bfvl	25.0
1m2od	1yzga	25.0
1ji4d	1dpsa	25.0
1g16a	1am4f	25.0
1eqwc	1xsob	25.0
1dp2a	1e0ca	25.0
1ckta	1o4xb	25.0
1lluh	1pl8d	24.9
1unpa	1fgya	24.8
1r1kd	1nq2a	24.8
1m5ua	4tmya	24.8
1jigd	1o9rc	24.8
1jhlh	1fvcc	24.8
1hbrd	1umoa	24.8
1hbra	1cp5a	24.8
1uluc	1x7ga	24.7
1nfie	1tr4a	24.7
1m7wd	1pcga	24.7
1eaga	1od1a	24.7
1d8za	1wi8a	24.7
1qrva	1j46a	24.6
1hysc	1bz7b	24.6
1b48a	1yq1b	24.6
2bfvl	1bfvh	24.5
1y6la	1yrva	24.5
1r5na	1xb2a	24.5
1coja	1kkcb	24.5
2fgib	1oiuc	24.4
1lp8a	1j1ma	24.4
1rzwa	1q7sa	24.3
1py5a	1jqhb	24.3
1pvva	1pg5a	24.3
1mp8a	1vzoa	24.3
1lxfc	1s6ja	24.3
1llna	1nioa	24.3
1f3mc	1sm2b	24.3
1rkga	1n46a	24.1
1qo3d	1sl5a	24.1
1pkgb	1t4ha	24.1
1eyxk	1liab	24.1
1byea	1n2ab	24.1
2ccyb	1gqad	24.0
1hyhc	1oc4b	24.0
1bkha	1jpdx	24.0
1akje	1ek3b	24.0
1xkza	1k6sa	23.9
1wg7a	1v5ua	23.9

1piwa	1r37b	23.9
1pd22	1u87a	23.9
1osha	1z5xu	23.9
1ioda	1rjha	23.9
1fdvd	1xseb	23.9
1v7qa	1tipb	23.8
1ty9b	1ci0b	23.8
1m63b	1jfja	23.8
1l6oc	1i92a	23.8
1kmha	1skye	23.8
1wf1a	1wf0a	23.7
1sb2b	1ypoa	23.7
1mb0a	1qmpb	23.7
1f3oa	1jj7a	23.7
1dxld	1aogb	23.7
1odke	1sq6a	23.6
1iasb	1k2pb	23.6
1g0nb	1h5qe	23.6
43c9g	1mhpx	23.5
1xkqb	1e3sb	23.5
1i3gh	1fgvl	23.5
1gyob	1qn1a	23.5
1fvsa	1tl5a	23.5
1a4pa	1k8ua	23.5
1sqna	1a52a	23.4
1sjxa	1ktrl	23.4
1rvfh	1jhll	23.4
1j2qh	1g0uf	23.4
1fb5a	1raic	23.4
1et6b	1ewca	23.4
1s05a	1bbhb	23.3
1rfdl	1ad9b	23.3
1pm5a	1k3xa	23.3
1ryph	1g0ul	23.2
1of2a	1a1mb	23.2
1it9h	1aqkl	23.2
1ew3a	1e5pc	23.2
1b6wa	1id3a	23.2
1wf2a	1wexa	23.1
1vyda	1lmsa	23.1
1yucb	1hj1a	23.0
1wf7a	1va8a	23.0
1vaea	1uf1a	23.0
1sw8a	1j55a	23.0
1pkqa	1cz8y	23.0
1jspb	1f68a	23.0
1fvub	1kza2	23.0
1xiwh	1f6ll	22.9
1wh1a	1wi2a	22.9
1shmd	1i3gl	22.9
1nbvh	1pg7y	22.9
1lv9a	1plfa	22.8
1a14h	1dvfc	22.8
1xg5a	1h5qd	22.7
1s0xa	1r20d	22.7
1rj4b	1x91a	22.7
1uufa	1f8fa	22.6
1ol0b	2dlfl	22.6

1kv6a	1pq9d	22.6
1ck0l	1rzfh	22.6
2afpa	1c3aa	22.5
1j1pl	1nmch	22.5
1iqta	1p1ta	22.5
1gm6a	1pboa	22.5
1f9ma	1t00a	22.5
1f94a	1qm7a	22.5
1ujta	1uena	22.4
1bqlh	1nfdd	22.4
1b2wh	1nfdg	22.4
1yb1a	1nxqa	22.3
1z3ka	1sprb	22.2
1yhue	1si4d	22.2
1xapa	1pcgb	22.2
1m0ub	2fheb	22.2
1k82d	1k3wa	22.2
1ac6b	1jv5b	22.2
1unra	1faoa	22.1
1i7zc	1bfog	22.1
1xu9c	1g0oc	22.0
1xqma	1yjfa	22.0
1wh3a	1v86a	22.0
1oe7b	2gsrb	22.0
1j05b	1kyok	21.9
1clol	1ggij	21.9
1kcvl	1fdlh	21.8
1f6ad	1ow0b	21.8
1axsl	1n8zb	21.8
1s3qc	1iesa	21.7
1oayh	1tvdb	21.7
1c16e	1qo3b	21.7
1p6ua	1oxbb	21.6
1i3ua	1vera	21.5
1h8ea	1bmfd	21.5
1f4pa	1b1ca	21.5
1dsfl	2bfvh	21.5
1pyta	1vjqa	21.4
1ypzg	1iqda	21.3
1ulsc	1bvrc	21.3
1xaea	1cv7a	21.2
1uwjb	1okza	21.2
1rgwa	1q7xa	21.2
1o1gh	1scmc	21.2
1gs4a	2bj4b	21.2
1nxkd	1s9ib	21.1
1hdma	1k8ib	21.1
1fb8a	1p6sa	21.1
1aqkh	1lilb	21.1
1o1gk	1dfkz	21.0
1dkfb	1uhla	21.0
1iru2	1fnti	20.9
1pq6a	1mzne	20.8
1oiva	1r4ad	20.8
1f2xl	1ac6a	20.8
1dfia	1gega	20.8
1whda	1uita	20.7
1v62a	1ufxa	20.7

1knoa	1rzga	20.6
1bvrb	1nfrb	20.6
1cu4l	1kcuh	20.4
1g91a	1qnkb	20.3
1fpub	1fota	20.3
1nxta	1qkka	20.2
1jv5a	1uach	20.2
1fl2a	1f6mf	20.2
1fe8l	1fe8h	20.2
1p1ea	1x8sa	20.0
1nr4d	1tvxa	20.0
1k68b	1ab6b	20.0
